# Evaluation of UK paediatric nephrology teams’ understanding, experience and perceptions of oral health outcomes and accessibility to dental care: a mixed-methods study

**DOI:** 10.1007/s00467-024-06292-x

**Published:** 2024-02-01

**Authors:** Christopher K. Wallace, Christopher R. Vernazza, Victoria Emmet, Nidhi Singhal, Vijaya Sathyanarayana, Yincent Tse, Greig D. Taylor

**Affiliations:** 1 Newcastle Dental Hospital, Newcastle Upon Tyne, Tyne and Wear UK; 2 School of Dental Sciences, Newcastle University, Newcastle Upon Tyne, UK; 3 Great North Children’s Hospital, Newcastle Upon Tyne, UK

**Keywords:** Dental, Paediatric nephrology, Oral health, Quality improvement, Training, Transplant, Inequalities

## Abstract

**Background:**

Oral health conditions are common in children and young people (CYP) with kidney disorders. There is currently limited literature on how confident paediatric nephrology teams feel to identify and manage oral health concerns for their patients.

**Method:**

An exploratory mixed-method survey was distributed across all 13 UK specialist paediatric nephrology centres with responses received from consultants, registrars, specialist nurses and special interest (SPIN) paediatricians.

**Results:**

Responses received from 109 multidisciplinary team members of 13/13 (100%) UK tertiary units. Ninety-two percent (*n* = 100) of respondents reported they had never received any training in oral health and 87% (*n* = 95) felt that further training would be beneficial to optimise care for patients and improve communication between medical and dental teams. Most respondents reported that they did not regularly examine, or enquire about, their patients’ oral health. Only 16% (*n* = 17) reported that all their paediatric kidney transplant recipients underwent routine dental assessment prior to transplant listing. Severe adverse oral health outcomes were rarely reported and only 11% (*n* = 12) of respondents recalled having a patient who had a kidney transplant delayed or refused due to concerns about oral infection. Seventy-eight percent (*n* = 85) felt that joint working with a dental team would benefit patients at their unit; however, 17% (*n* = 18) felt that current infrastructure does not currently support effective joint working.

**Conclusions:**

Across the UK, paediatric kidney health professionals report lack of confidence and training in oral health. Upskilling subspecialty teams and creating dental referral pathways are recommended to maximise oral health outcomes for CYP with kidney diseases.

**Graphical abstract:**

**Figure:**
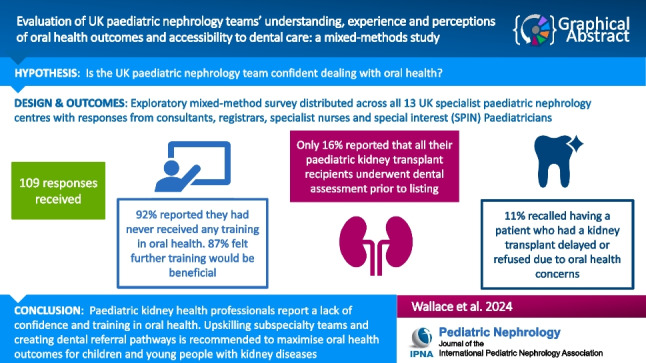
A higher resolution version of the Graphical abstract is available as Supplementary information

**Supplementary Information:**

The online version contains supplementary material available at 10.1007/s00467-024-06292-x.

## Introduction

In 2021, the World Health Organisation placed oral health back on the global health agenda with the publication of the resolution on oral health [1]. This includes high resource settings such as the UK where one in four (23%) of 5-year-olds had experience of dental decay in 2019 [2]. Oral health conditions are common in children and young people (CYP) with kidney disorders, for example 90% (*n* = 97) of paediatric kidney transplant recipients (KTRs) required some form of additional dental management during 4-year follow-up [3]. Immunocompromised nephrology patients are at increased risk of opportunistic, severe or even life-threatening oral infections [4, 5]. Kidney disease in childhood can cause developmental defects of enamel (DDE) which can lead to sensitivity, pain and poor aesthetics, negatively impacting on quality of life [3, 6–11]. A case–control study, including 256 Venezuelan paediatric patients (160 with tubulopathies and 96 healthy controls), reported that 90 renal patients had DDE compared to 28 controls, which conforms with known literature that renal disease can cause issues with tooth development [11]. Although less common, 1–2% of paediatric patients with KTR will develop post-transplant lymphoproliferative disease (PTLD), within 5 years, which may present with oral soft tissue changes, neck or parotid lumps [12]. Other oral soft tissue changes may include halitosis, dysgeusia, xerostomia, gingivitis, oral ulcerations, stomatitis, glossitis and leukoplakia [13, 14]. Medications used by patients with kidney disease can directly impact oral health. Drug-induced gingival overgrowth (DIGO), caused by medications such as ciclosporin and nifedipine, can adversely affect periodontal health and aesthetics [3, 13, 15] whilst immunosuppressants increase the risk of oral cancer and lymphoma [14, 16, 17].

Optimising oral health and ensuring regular dental care for CYP with kidney conditions are therefore imperative to prevent complications, especially in those who are immunocompromised [1]. In the UK, general dental practitioners (GDPs) undertake routine dental care for children, free on the National Health Service (NHS). More complex patients are referred to specialist paediatric dental services. Unfortunately, access to GDP services in the UK has recently become more limited with few practices currently providing care for paediatric patients [18, 19]. Given this access problem, initiatives such as ‘Mini Mouth Care Matters’ aim to bridge this gap by empowering non-dental healthcare professionals to incorporate oral healthcare into their routine care for paediatric inpatients and signpost those most at need [20]. National ‘Delivering Better Oral Health’ preventative guidelines are also freely available for healthcare teams to access [21]. However, current evidence reports that whilst paediatricians frequently encounter patients with dental issues, they may lack the confidence to recognise common dental conditions or reinforce preventative advice [22–28]. To our knowledge, there is limited literature on how confident paediatric kidney teams feel to do this for their patients.

The aim of this study is to evaluate UK nephrology team members’ understanding, experience and perceptions of oral health outcomes and accessibility to dental care.

## Method

This exploratory mixed-methods study received favourable ethical approval from Newcastle University Ethics Committee (Ref: 24983/2022).

### Context

There are 13 specialist units in the UK providing care for CYP with kidney diseases. Members of the nephrology team include consultants, specialty training registrars (STRs) and specialist nurses. In addition, these children may be cared for more locally by special interest (SPIN) paediatricians who have completed additional training within paediatric nephrology [29].

### Questionnaire design

An online questionnaire was developed using existing literature of oral health surveys of medical professionals, with questions being adapted specifically for CYP with kidney diseases. Piloting was initially undertaken for content, construct and face validity with members of the research team. Further piloting with paediatric dental and nephrology professionals was carried out using ‘speak aloud’ interviews, which supported the validity of the questions and their alignment to the intended aims of the study. Following this piloting, minor amendments were made to the layout of the questionnaire only. The questionnaire was created and delivered using survey software (Online Surveys©, Jisc, Bristol, UK) [30].

### Questionnaire content

The 17-item survey covered four sections using a mixture of open- and closed-questions (including Likert scales). Section 1 collected respondent demographics. Section 2 explored respondents’ subjective perceptions of the burden of oral disease for CYP with kidney disease. Section 3 explored the participants’ confidence and training at identifying and managing oral health concerns. The final section evaluated perceptions on patient access to dental care. The full survey is available as a Supplementary file.

### Sample

All paediatric nephrology consultants, STRs, SPIN paediatricians and specialist nurses in the UK were invited. Each unit was contacted to ascertain the total number of each team member in that unit. A total possible sample size of 268 was calculated.

### Questionnaire distribution

The link to the online questionnaire was disseminated to each unit, with colleagues being asked to forward to all other relevant members within their unit. In addition, the link was shared via the national STRs and SPIN paediatricians’ groups and also included in the British Association of Paediatric Nephrology (BAPN) newsletter. Data was collected between 26th October 2022 and 26th January 2023, with reminder emails to encourage participation.

### Data analyses

Collected data were cleaned, validated and transferred into IBM® SPSS® Statistics [31] for Windows and Microsoft Excel [32]. Descriptive statistics were used to explain participant demographics. Data was deemed to be not normally distributed by the Kolmogorov–Smirnov test. Comparisons between the medical and nursing staff groups, created for sub-group analyses, were completed using the chi-squared test for categorical variables and Spearman’s correlation for ordinal data. Free-text comments were assessed using the principles of thematic analysis [33] by two members of the research team (CW and GT) independently. Evolving themes were discussed with the wider research team before consensus was reached.

## Results

The response rate was 40.7% (*n* = 109) with representation from all 13 specialist UK units. A breakdown of participant demographics is shown in Table 1. Two groups were formed for sub-group analyses: medics (consultants, STRs and SPIN paediatricians; *n* = 53) and nurses (*n* = 56).

**Table 1:** Participant demographics

	Number of responses	Estimated total in UK workforce	Response rate
Consultants	35	77	45.5%
STR	10	18	55.6%
Specialist nurse	56	92	60.9%
SPIN paediatrician	8	81	9.9%
Total	109	268	40.7%

The median number of years worked for a specialist nurse was 16.5 years (IQR: 8.5–22.75) and for medics 8 years (IQR: 4–22). Most respondents worked with more than one group of patients, with 66% (*n* = 72), 61% (*n* = 66), 57% (*n* = 62), 56% (*n* = 61) and 56% (*n* = 61) reporting to have worked with chronic kidney disease (CKD), kidney transplant recipients (KTR), haemodialysis, peritoneal dialysis and nephrotic syndrome patients, respectively.

Participants’ self-reported experience of the frequency of dental problems encountered is summarised in Table 2. Unsurprisingly, poor dental aesthetics, a negative oral health-related quality of life and DIGO were the most common oral health complications reported, whilst more serious oral health conditions such as sepsis or oral cancer were virtually never reported. Twelve respondents reported a delay or refusal of a kidney transplant due to concerns about oral health, with a median of 1 (IQR: 1–2.75; max. 10 patients) patient affected. Of those who managed KTR patients, 27 respondents reported observing a swelling or infection from a dental cause post-transplant, with a median of 2 (IQR: 1–3; max. 6) patients affected.

**Table 2:** Frequency of dental problems encountered, *n* = 109 (%)

	Less than annually or never	Quarterly to annually	Monthly or more frequent
Poor dental aesthetics	26 (23.9%)	42 (38.5%)	41 (37.6%)
Subjective negative oral health-related quality of life (OHRQoL)	32 (29.4%)	57 (52.3%)	20 (18.3%)
Drug-induced gingival overgrowth (DIGO)	47 (43.1%)	49 (45.0%)	13 (11.9%)
Toothache	52 (47.7%)	53 (48.6%)	4 (3.7%)
Gum swelling (dental cause)	68 (62.4%)	37 (33.9%)	4 (3.7%)
Facial swelling (dental cause)	88 (80.7%)	21 (19.3%)	0
Sepsis (dental cause)	100 (91.7%)	9 (8.3%)	0
Oral post-transplant lymphoproliferative disorder (PTLD)	108 (99.1%)	1 (0.9%)	0
Oral cancer	109 (100%)	0	0

Participants’ confidence in oral health is summarised by Table 3. There was no statistically significant difference between nurses and medics on how frequently they examined patients’ oral health (*p* = 0.764). Similarly, no statistically significant differences were noted in confidence between nurses and medics with respect to diagnosing caries (*p* = 0.154), developmental defects of enamel (*p* = 0.064) or DIGO (*p* = 0.314). There were no correlations between how frequently medics and nurses reported examining their patients’ oral health and how confident they felt at diagnosing caries (*p* = 0.164) or DIGO (*p* = 0.105).

**Table 3:** Confidence and practices related to oral health (*n* = 109)

		Strongly disagree or disagree	Neither agree nor disagree	Agree or strongly agree	
Self-confidence					
I feel confident identifying dental caries	Nurse group (*n* = 56)	43	12	1	*p* = 0.154
	Medic group (*n* = 53)	32	8	13	
I feel confident identifying developmental defects of enamel (DDE) such as hypomineralisation or hypoplasia	Nurse group (*n* = 56)	51	4	1	*p* = 0.064
	Medic group (*n* = 53)	41	6	6	
I feel confident identifying drug-induced gingival overgrowth (DIGO)	Nurse group (*n* = 56)	30	9	17	*p* = 0.314
	Medic group (*n* = 53)	8	16	29	
I feel knowledgeable enough to counsel parents regarding home dental care for their children	Nurse group (*n* = 56)	17	11	28	*p* = 0.368
	Medic group (*n* = 53)	21	19	13	
Self-practice					
I feel my team’s patients should receive a dental assessment as part of the transplant work-up process	Nurse group (*n* = 56)	1	13	42	*p* = 0.078
	Medic group (*n* = 53)	2	7	44	
I regularly examine my patients’ oral health	Nurse group (*n* = 56)	28	22	6	*p* = 0.764
	Medic group (*n* = 53)	27	15	11	
There is inadequate time during consultations to address oral health	Nurse group (*n* = 56)	16	18	22	*p* = 0.234
	Medic group (*n* = 53)	11	16	26	

Most respondents (92% (*n* = 100)) had not received any dedicated teaching on oral health for CYP with kidney diseases. For those who had (*n* = 9), 8 had attended a single dental presentation (of whom 6 felt this training was satisfactory) and 1 was previously a dental nurse and hence had received more comprehensive training. The majority of respondents (87% (*n* = 95)) felt further specific training in oral health would be beneficial. Most respondents were unaware of ‘Delivering Better Oral Health Guidelines’ [21] (82% (*n* = 89)) and ‘Mini Mouth Care Matters’ (85% (*n* = 93)), respectively [20].

Most respondents (*n* = 89) reported a less than monthly encounter of patients struggling to access dental care, with 17% (*n* = 19) reporting they had never had a patient report difficulty accessing dental care. Seventy-three percent (*n* = 80) of respondents either did not know how many of their kidney transplant recipients received dental assessment prior to transplant or reported that less than half of their KTRs received this. However, 16% (*n* = 17) reported that all their KTRs received dental assessment prior to transplant. As shown in Table 4, a varying response to ease of access to specialist paediatric dentistry services was reported. There was no statistically significant difference between nurses and medics on how confident they were in referring a patient to specialist paediatric dentistry services (*p* = 0.577) or how easily they felt their patients could access these services, when required (*p* = 0.403).

**Table 4:** Participants’ perceptions of access to dental services (*n* = 109)

		Disagree or strongly disagree	Neither agree nor disagree	Agree or strongly agree
I am confident how to refer my patients to specialist paediatric services	Nurse group (*n* = 56)	16	15	25
	Medic group (*n* = 53)	6	3	44
I feel I can easily access specialist paediatric dentistry advice when needed	Nurse group (*n* = 56)	17	11	28
	Medic group (*n* = 53)	6	13	34
Our team has a well-established pathway for referring patients for specialist paediatric dentistry assessments	Nurse group (*n* = 56)	20	18	18
	Medic group (*n* = 53)	10	19	24
I think joint working with dental team would be feasible at my unit	Nurse group (*n* = 56)	8	19	29
	Medic group (*n* = 53)	10	14	29
I think joint working with a dental team would benefit patients at my unit	Nurse group (*n* = 56)	1	11	44
	Medic group (*n* = 53)	2	10	41

Most respondents (78%, *n* = 85) agreed or strongly agreed that joint working with a dental team would benefit patients at their unit; however, 17% (*n* = 18) disagreed or strongly disagreed that joint working would be feasible at their unit. Most respondents reported that all barriers to care, as illustrated in Table 5, represented a moderate or significant burden for their patients, with waiting lists for an NHS dentist being perceived as the greatest barrier to care.

**Table 5:** Considering your patients as a whole, please select how much you feel the following barriers apply to their ability to access dental care (*n* = 109)

	Not a barrier for my patients	A mild barrier for my patients	A moderate barrier for my patients	A significant barrier for my patients
Burden of existing healthcare appointments	1	17	55	36
Geographical isolation	19	29	46	15
Cost of travel to dental appointments	7	26	50	26
Patients’ general health	17	34	49	9
Waiting lists for an NHS primary care dentist	3	9	37	60
Difficult accessing a specialist in paediatric dentistry	15	30	39	25

Thematic analysis [33] of respondents’ free-text comments (*n* = 82) identified two main themes:

1. Identification and optimisation of oral care for patients
  ‘This would help us identify any issues that patients may have and refer appropriately. We could also then support families and patients more.’ Specialist nurse, 7 years’ experience.
  ‘So a clinician is confident in identifying common dental problems in CYP and able to refer them for specialist input. Assessing dental health is mandatory especially for children with kidney conditions, not only as part of general wellbeing but a prerequisite before exposing them to immunosuppressive agents post-transplant.’ Paediatric nephrology consultant, 6 years’ experience.
  ‘…this is not something I know very much about and currently wouldn’t be confident supporting patients with ongoing dental health needs.’ Specialist nurse, 10 years’ experience.
  ‘[It would help] knowing what to look out for, to potentially pick up issues earlier…’ Specialist nurse, 19 years’ experience.
  ‘While happy to provide general guidance, lack of NHS dentistry should not be absorbed by paediatric nephrology services. I should be able to say, “Please see your dentist!”’ Paediatric nephrology consultant, 7 years’ experience.
2. Communication, education and signposting between professional teams
  ‘Oral health is important for all children, as we have regular contact it would be good to know that we can advise correctly and can signpost accordingly where there are concerns.’ Specialist nurse, 18 years’ experience.
  ‘It would be useful for training for dentists on impact on oral health and to understand [there are] often no risks to treatment to avoid treatment delays in letters asking for medical contraindications to treatment.’ Paediatric nephrology consultant, 8 years’ experience.
  ‘Oral health is often not prioritised during work up, despite it being a required element of our work up protocol, and further education may highlight its importance to professionals and also aid health promotion.’ Specialist nurse, 7 years’ experience.
  ‘Mainly for identification and signposting to oral health experts; not in order to manage myself.’ SPIN paediatrician, 10 years’ experience.

## Discussion

To our knowledge, this is the first study to comprehensively explore healthcare staff’s understanding, experience and perceptions of oral health outcomes and accessibility to dental care in one subspecialty across a whole healthcare system. Overall, there appears to be a general lack of training, confidence and understanding, but a desire to better the oral health of their patients.

These learning needs are attainable with modest resource as there are only a small number of presentations that are frequently encountered. Paediatric nephrology team members have a role in advocating for good oral health of their patients as well as signposting any patients with oral health issues to relevant dental professionals. Identification of common oral diseases is lacking and could be considered outside the scope of practice of a paediatric nephrologist; however, similar skills already exist as there is a high degree of confidence and practice in areas such as identifying (DIGO) [15]. Fortunately, serious adverse oral health outcomes such as sepsis from a dental source and/or the presence of an oral cancer were rarely reported, although this likely reflects the low prevalence of these conditions in children in general [34]. Ultimately, this study suggests that nephrology teams are already considering the oral cavity, often a known barrier with other medical professional teams [22–28], but require some additional training to identify more common dental issues. Building on these skills would permit them to act as vital safety nets and signpost appropriately for this patient cohort.

Our study has identified service gaps. Few paediatric KTRs receive pre-transplant dental assessment although this has not been mandated by national guidance. However, given the rigorous treatment required [13, 35], it would seem reasonable as our survey showed that some patients had transplants delayed for dental treatment. There is clear guidance for paediatric oncology [36] and cardiac [37] patients to have an oral screen prior to any treatment or transplant. Development of national oral health standards could help reduce variability in care and ensure adequate commissioning of dental services, with this approach already being successfully implemented for CYP with congenital cardiac and oncological diseases [36, 37].

There are clear benefits if both dental and nephrology teams work together to co-ordinate screening and/or treatment appointments to overcome the apparent geographical and financial barriers that parents face. Defining clear referral pathways [13, 38] and providing appropriate lines of communication between the teams will support this joint effort. An example of such a pathway has recently been developed in the North East of England [38]. It is acknowledged that this may be more difficult in areas with reduced paediatric dentistry provision [39]. It would be reasonable that in such circumstances, primary dental care providers could undertake a dental screen. Unfortunately, there is currently a widely acknowledged NHS dental access crisis [18, 19]; however, there appeared to be very few cases where nephrology teams noted that their patients struggled to access dental care. This is perhaps due to most respondents not routinely asking their patients about accessing dental care and may not therefore be a true reflection of the problems these patients may face.

Understanding the knowledge and perceptions of oral health outcomes amongst paediatric nephrology teams is not just relevant to the UK. Despite the variation in global prevalence [40], a proportion of patients with CKD across different countries will exhibit dental disease. The realistic impacts on these patients would be similar to those children with CKD in the UK, thus acknowledging the relevance of these findings internationally. It could be expected that each country’s healthcare system could influence these findings. The NHS in the UK is a publicly funded healthcare system with no cost to the patient/parent at point of delivery for medical and dental care. This makes it easier for nephrology teams to refer patients with CKD for oral health screening and dental management. However, this is not true of all healthcare systems as significant variation in the levels of co-payment for managing children with CKD and/or oral disease will exist [41]. Co-payment charges, or limitations in insurance coverage, could make it challenging for nephrology teams to refer for oral issues, with parents being forced to focus on medical care needs only. As a result, in these situations, upskilling nephrology teams to provide basic preventive oral health care messages could have demonstrable benefits to this patient cohort. Hypothetically, this could improve a patient’s quality of life, have less impact on renal outcomes and treatments and potentially reduce the financial burden to the family.

The strength of this unique mixed-methods study was comprehensive coverage from all UK specialist teams, and it was truly multidisciplinary. However, it is acknowledged that this study was bespoke to the UK’s NHS healthcare system. Replicating this study in other countries would permit greater understanding of the oral/dental interface, at a global level, whilst appreciating the training needs for nephrology teams pertinent to their patient cohort and the health system they work within. The study design was limited as some responses may be overestimated given that multiple members from the same unit may report the same patient. The questionnaire was intended to subjectively assess respondents’ perceptions, rather than objectively. However, it is acknowledged this could cause some bias; for example, the definitions of a mild, moderate and severe barrier found within Table 5 are open to interpretation. In addition, respondents’ perceptions of the burden of oral disease for CYP with kidney disease were assessed subjectively. Findings from this study would indicate the benefit of formally assessing the OHRQoL of children and young people with CKD using a validated patient-reported outcome measure. Similarly, thematic analysis was only completed on the free-text comments. Future research should focus on fully exploring these views using qualitative methods.

## Conclusion

Across the UK, paediatric nephrology professionals report lack of confidence in the understanding the impact oral health can have for their patients. Upskilling subspecialty teams and creating dental referral pathways are recommended to maximise oral health outcomes, prevent complications and improve the overall outcome for patients with kidney diseases.

### Supplementary Information

Below is the link to the electronic supplementary material.

Graphical abstract (PPTX 104 KB)

Supplementary file1 (PDF 548 KB)

## Data Availability

The data that support the findings of this study are available from the corresponding author, Greig Taylor (Greig.Taylor@newcastle.ac.uk), upon reasonable request.

## References

[1] Lamster IB (2021). The 2021 WHO resolution on oral health. Int Dent J.

[2] Public Health England (2019) National Dental Epidemiology Programme for England: oral health survey of 5-year-olds 2019. Available at: https://www.gov.uk/government/statistics/oral-health-survey-of-5-year-old-children-2019 (Accessed 11 October 23)

[3] Farge P, Ranchin B, Cochat P (2006). Four year follow-up of oral health surveillance in renal transplant children. Pediatr Nephrol.

[4] Greenwood M, Meechan JG, Bryant DG (2003). General medicine and surgery for dental practitioners. Part 7: renal disorders. BDJ.

[5] Shiboski CH, Kawada P, Golinveaux M (2009). Oral disease burden and utilization of dental care patterns among pediatric solid organ transplant recipients. J Public Health Dent.

[6] Al Nowaiser A, Roberts GJ, Trompeter RS (2003). Oral health in children with chronic renal failure. Pediatr Nephrol.

[7] Koch MJ, Buhrer R, Pioch T (1999). Enamel hypoplasia of primary teeth in chronic renal failure. Pediatr Nephrol.

[8] Nunn J, Sharp J, Lambert HJ (2000). Oral health in children with renal disease. Pediatr Nephrol.

[9] Sezer B, Kaya R, Dokumacıgil NK (2023). Assessment of the oral health status of children with chronic kidney disease. Pediatr Nephrol.

[10] Silva TMC, Alves LAC, Garrido D (2019). Health and oral health-related quality of life of children and adolescents with chronic kidney disease: a cross-sectional study. Qual Life Res.

[11] Acosta de Camargo MG, Oliveros DJ, Coronel V et al (2018) Association between oral finding and renal disease among pediatric patients in Venezuela. Rev ADM 75:71–79. (English abstract) (https://www.medigraphic.com/pdfs/adm/od-2018/od182c.pdf)

[12] Mynarek M, Hussein K, Kreipe HH (2014). Malignancies after pediatric kidney transplantation: more than PTLD?. Pediatr Nephrol.

[13] Velan E, Sheller B (2021). Oral health in children with chronic kidney disease. Pediatr Nephrol.

[14] Noriega S, Acosta de Camargo MG (2017) Oral manifestations in pediatric patients with lupus nephritis. KIRU 14:58–67. (English abstract) 10.24265/kiru.2017.v14n1.08

[15] Heasman P, Hughes F (2014). Drugs, medications and periodontal disease. BDJ.

[16] Petti S, Polimeni A, Berloco PB (2013). Orofacial diseases in solid organ and hematopoietic stem cell transplant recipients. Oral Dis.

[17] Francis A, Johnson DW, Craig JC (2017). Incidence and predictors of cancer following kidney transplantation in childhood. Am J Transplant.

[18] British Dental Association (2022) NHS dentistry at tipping point, as BBC reveal true extent of access crisis. https://bda.org/news-centre/press-releases/Pages/nhs-dentistry-at-a-tipping-point.aspx (Accessed 25 July 2023)

[19] British Broadcasting Corporation (2022) Full extent of NHS dentistry shortage revealed by far-reaching BBC research. https://www.bbc.co.uk/news/health-62253893 (Accessed 25 July 2023)

[20] Health Education England (2022) Mouth care matters. https://mouthcarematters.hee.nhs.uk/about-the-programme/children/index.html (Accessed 24 August 2023)

[21] Department of Health and Social Care (2021) Delivering better oral health. https://www.gov.uk/government/publications/delivering-better-oral-health-an-evidence-based-toolkit-for-prevention (Accessed 21 August 2023)

[22] Dickson-Swift V, Kenny A, Gussy M (2020). The knowledge and practice of pediatricians in children’s oral health: a scoping review. BMC Oral Health.

[23] Lewis CW, Grossman DC, Domoto PK (2000). The role of the pediatrician in the oral health of children: a national survey. Pediatrics.

[24] Aburahima N, Hussein I, Kowash M (2020). Assessment of paediatricians’ oral health knowledge, behaviour, and attitude in the United Arab Emirates. Int J Dent.

[25] Hadjipanayis A, Grossman Z, Del Torso S (2018). Oral health training, knowledge, attitudes and practices of primary care paediatricians: a European survey. Eur J Pediatr.

[26] Balaban R, Menezes Aguiar C, Silva Araujo AC (2012). Knowledge of paediatricians regarding child oral health. Int J Paediatr Dent.

[27] Koirala A, O’Connor E, Widmer R (2019). Oral health care: the experience of Australian paediatricians. J Paediatr Child Health.

[28] Kalkani M, Ashley P (2013). The role of paediatricians in oral health of preschool children in the United Kingdom: a national survey of paediatric postgraduate specialty trainees. Eur Arch Paediatr Dent.

[29] Royal College of Paediatrics and Child Health (2023). Special interest (SPIN) modules - application guidance. Available at: https://www.rcpch.ac.uk/education-careers/apply-paediatrics/SPIN-modules (Accessed 7 May 2023)

[30] Jisc. Online Surveys© (2023) Available at: https://www.onlinesurveys.ac.uk/ (Accessed 1 Dec 2022)

[31] IBM®. SPSS Statistics® (2023) Available at: https://www.ibm.com/products/spss-statistics (Accessed 24 Sept 2023)

[32] Microsoft Corporation (2018) Microsoft Excel. Available at: https://office.microsoft.com/excel (Accessed 24 Sept 2023)

[33] Braun V, Clarke V (2006). Using thematic analysis in psychology. Qual Res Psychol.

[34] PDQ Pediatric Treatment Editorial Board (2022). Childhood oral cavity cancer treatment (PDQ®): health professional version. In: PDQ cancer information summaries. Bethesda (MD): National Cancer Institute (US) 29337482

[35] Dudley J, Christian M, Andrews A (2021). Clinical practice guidelines standardisation of immunosuppressive and anti-infective drug regimens in UK paediatric renal transplantation: the harmonisation programme. BMC Nephrol.

[36] The Royal College of Surgeons of England & The British Society of Disability and Oral Health (2018) The oral management of oncology patients requiring radiotherapy, chemotherapy and/or bone marrow transplantation. Available at: https://www.rcseng.ac.uk/dental-faculties/fds/publications-guidelines/clinical-guidelines/ (Accessed 3 August 2023)

[37] Hughes S, Balmer R, Moffat M (2019). The dental management of children with congenital heart disease following the publication of paediatric congenital heart disease standards and specifications. Br Dent J.

[38] Wallace CK, Henesy F, Atkinson J et al (2023) Dental pathway for paediatric nephrology patients: a quality improvement project. Int J Paediatr Dent 3(Supp 2) 10.1111/ipd.13107

[39] Jo O, Kruger E, Tennant M (2021). Dental specialist workforce and distribution in the United Kingdom: a specialist map. Br Dent J.

[40] Bernabe E, Marcenes W, Hernandez CR (2020). Global, regional, and national levels and trends in burden of oral conditions from 1990 to 2017: a systematic analysis for the Global Burden of Disease 2017 Study. J Dent Res.

[41] Bello AK, Levin A, Lunney M (2019). Status of care for end stage kidney disease in countries and regions worldwide: international cross sectional survey. BMJ.

