# Supplementary material for: Evaluation of UK paediatric nephrology teams’ understanding, experience and perceptions of oral health outcomes and accessibility to dental care: a mixed-methods study
Source: Pediatr Nephrol. 2024 Feb 1;39(7):2131–8. doi: 10.1007/s00467-024-06292-x (PMC11147922; doi:10.1007/s00467-024-06292-x)
Supplement: Supplementary file 1 — Graphical abstract (PPTX 104 KB) [file 467_2024_6292_MOESM1_ESM.pptx]

## Slide 1
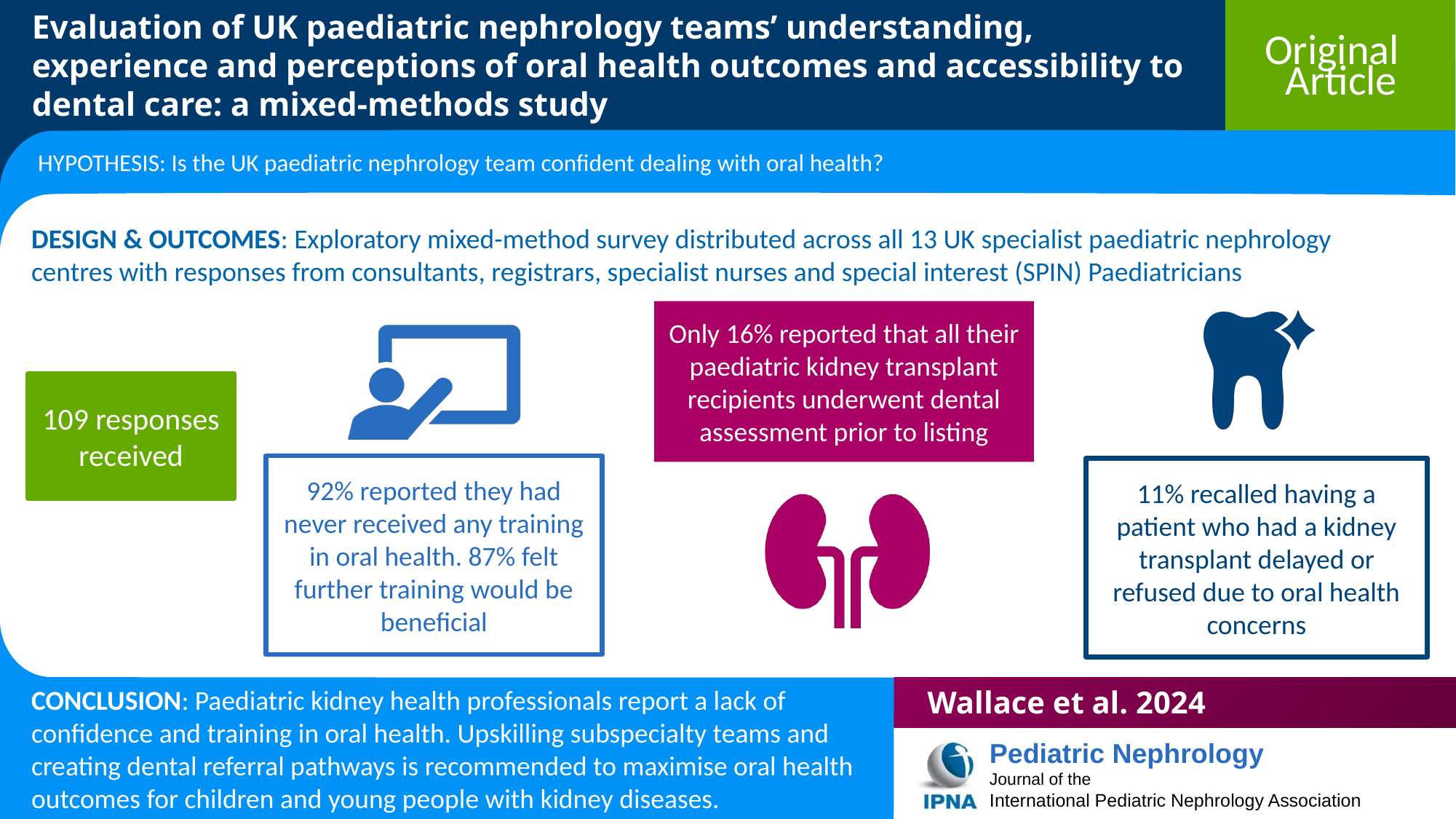

Evaluation of UK paediatric nephrology teams’ understanding, experience and perceptions of oral health outcomes and accessibility to dental care: a mixed-methods study
HYPOTHESIS: Is the UK paediatric nephrology team confident dealing with oral health?
DESIGN & OUTCOMES: Exploratory mixed-method survey distributed across all 13 UK specialist paediatric nephrology centres with responses from consultants, registrars, specialist nurses and special interest (SPIN) Paediatricians
Only 16% reported that all their paediatric kidney transplant recipients underwent dental assessment prior to listing
109 responses received
92% reported they had never received any training in oral health. 87% felt further training would be beneficial
11% recalled having a patient who had a kidney transplant delayed or refused due to oral health concerns
CONCLUSION: Paediatric kidney health professionals report a lack of confidence and training in oral health. Upskilling subspecialty teams and creating dental referral pathways is recommended to maximise oral health outcomes for children and young people with kidney diseases.
Wallace et al. 2024
