# Supplementary material for: Evaluation of UK paediatric nephrology teams’ understanding, experience and perceptions of oral health outcomes and accessibility to dental care: a mixed-methods study
Source: Pediatr Nephrol. 2024 Feb 1;39(7):2131–8. doi: 10.1007/s00467-024-06292-x (PMC11147922; doi:10.1007/s00467-024-06292-x)
Supplement: Supplementary file 2 — Supplementary file1 (PDF 548 KB) [file 467_2024_6292_MOESM2_ESM.pdf]

Evaluation of UK Paediatric Nephrology Teams’ understanding, experience and perceptions of oral health outcomes and accessibility to dental care

Page 1: Introduction

You are being invited to participate in a cross-sectional survey entitled “Evaluation of UK Paediatric Nephrology Teams’ understanding, experience and perceptions of oral health outcomes and accessibility to dental care”.

This is a UK-wide survey being led by the Newcastle University School of Dental Sciences and the Great North Children's Hospital. Favourable ethical approval has been obtained from Newcastle University (24983/2022).

The aim of the survey is to evaluate paediatric nephrology consultants’, registrars’ and specialist nurses’ experience of adverse oral health outcomes in Children and Young People (CYP) with kidney disease. Furthermore, it will evaluate your perceptions of how readily you feel your patients can access dental care and how confident you feel at recognising and escalating relevant oral health concerns in your patient group.

We estimate that the survey will take no more than **10 minutes** to complete. Participation is voluntary.

1. Do you consent to take part in this survey? ✱ *Required*

○ Yes

Page 2: Section 1- Demographics

2. Please select your job role ✱ *Required*

- ○ Paediatric Nephrology Consultant
- ○ Paediatric Nephrology Registrar
- ○ Paediatrician with a special interest in nephrology
- ○ Staff Grade (SAS)
- ○ Specialist Nurse
- ○ Other

2.a. If you selected Other, please specify:

3. Which unit do you currently work at? ✱ *Required*

- ○ Belfast
- ○ Birmingham
- ○ Bristol
- ○ Cardiff
- ○ Evelina London Children's Hospital
- ○ Glasgow
- ○ Great Ormond Street
- ○ Leeds
- ○ Liverpool
- ○ Manchester
- ○ Newcastle
- ○ Nottingham
- ○ Southampton
- ○ Other

3.a. If you selected Other, please specify:

4. Which patient groups do you primarily care for? (Please select all that apply) ✱ *Required*

Please select at least 1 answer(s).

- ☐ Chronic Kidney Disease (CKD)
- ☐ Nephrotic Syndrome
- ☐ Post-transplant
- ☐ Haemodialysis
- ☐ Peritoneal Dialysis

5. How many years have you been working within the field of paediatric nephrology?
✱ *Required*

Please enter a whole number (integer).

The number should be 0 or greater.

Your answer should be no more than 2 characters long.

Page 3: Section 2- Experience of Adverse Oral Health Outcomes

6. Please estimate how frequently you encounter the following problems when working with Children and Young People (CYP) with kidney disease

	✱ *Required*						
	Never	Less than Annually	Annually	Quarterly	Monthly	Weekly	Daily
Toothache	○	○	○	○	○	○	○
Gum swelling from a suspected or known dental cause	○	○	○	○	○	○	○
Facial swelling from a suspected or known dental cause	○	○	○	○	○	○	○
Sepsis from a suspected or known dental cause	○	○	○	○	○	○	○
Poor dental aesthetics	○	○	○	○	○	○	○
Drug-induced gingival overgrowth	○	○	○	○	○	○	○
Poor oral health having a negative impact on patients' quality of life	○	○	○	○	○	○	○
Oral Cancer	○	○	○	○	○	○	○
Oral presentation of post-transplant lymphoproliferative disorder	○	○	○	○	○	○	○

7. Have you had a patient who has had a kidney transplant delayed or refused due to concern about their oral health? ✱ *Required*

- ○ Yes
- ○ No

7.a. Approximately how many patients has this been the case for?

Please enter a whole number (integer).

The number should be 1 or greater.

Your answer should be no more than 4 characters long.

8. Have you had a kidney transplant recipient suffer swelling or infection from a dental cause after transplant? ✱ *Required*

- ○ Yes
- ○ No
- ○ I have never worked with the post-transplant patient group

8.a. Approximately how many patients has this been the case for?

Please enter a whole number (integer).

The number should be 1 or greater.

Your answer should be no more than 4 characters long.

Page 4: Section 3- Confidence and Practices Related to Oral Health

9. Please select how much you agree or disagree with the following statements

	✱ *Required*				
	Strongly Disagree	Disagree	Neither agree nor disagree	Agree	Strongly Agree
I feel my team's patients should receive a dental assessment as part of the transplant work-up process	○	○	○	○	○
I regularly examine my patients' oral health	○	○	○	○	○
There is inadequate time during consultations to address oral health	○	○	○	○	○
I feel confident identifying dental caries	○	○	○	○	○
I feel confident identifying Developmental Defects of Enamel (DDE) such as hypomineralisation or hypoplasia	○	○	○	○	○
I feel confident identifying Drug-Induced Gingival Overgrowth (DIGO)	○	○	○	○	○
I feel knowledgeable enough to counsel parents regarding home dental care for their children	○	○	○	○	○

10. Approximately how many of your kidney transplant patients receive a dental assessment prior to transplant? ✱ *Required*

- ◯ I don't know
- ◯ None- we do not currently have a dedicated pathway for this
- ◯ < 10%
- ◯ 10-25%
- ◯ 25-50%
- ◯ 50-75%
- ◯ 75-99%
- ◯ All- 100%

11. During your postgraduate education, have you received any dedicated teaching on oral health in CYP with kidney diseases? ✱ *Required*

- ◯ Yes
- ◯ No

11.a. How would you rate the level of any training you have received?

- ◯ Wholly Inadequate
- ◯ Inadequate
- ◯ Satisfactory
- ◯ More than satisfactory
- ◯ Excellent

11.b. Please briefly describe any training you have received ✱ *Required*

➕ More info

Your answer should be no more than 400 characters long.

12. Do you think further training in oral health would be beneficial? ✱ *Required*

- ○ Yes
- ○ No

12.a. Why so?

13. Are you aware of the Department of Health Document "Delivering Better Oral Health"? ✱ *Required*

- ○ Yes
- ○ No

14. Are you aware of Health Education England's "Mini Mouth Care Matters"? ✱ *Required*

- ○ Yes
- ○ No

Page 5: Section 4- Access to Dental Care

15. Do you routinely ask your patients whether they are accessing regular dental care? ✱ *Required*

- ○ Yes
- ○ No

16. Approximately how often do you encounter patients who report difficulty accessing dental care? ✱ *Required*

- ○ Never
- ○ Annually
- ○ Quarterly
- ○ Monthly
- ○ Weekly
- ○ Daily

17. Please select how much you agree or disagree with the following statements

	✱ *Required*				
	Strongly Disagree	Disagree	Neither Agree nor Disagree	Agree	Strongly Agree
I am confident how to refer my patients to specialist paediatric dentistry services	○	○	○	○	○
I feel I can easily access specialist paediatric dentistry advice when needed	○	○	○	○	○
Our team has a well-established pathway for referring patients for specialist paediatric dentistry assessments	○	○	○	○	○
I think having joint working with a dental team would be feasible at my unit	○	○	○	○	○
I think having joint working with a dental team would benefit my patients at my unit	○	○	○	○	○

18. Considering your patients as a whole, please select how much you feel the following barriers apply to their ability to access dental care

	✱ *Required*			
	Not a barrier for my patients	A mild barrier for my patients	A moderate barrier for my patients	A significant barrier for my patients
Burden of existing healthcare appointments	○	○	○	○
Geographical isolation	○	○	○	○
Cost of travel to dental appointments	○	○	○	○
Patients' general health	○	○	○	○
Waiting lists for an NHS primary care dentist	○	○	○	○
Difficulty accessing a specialist in paediatric dentistry	○	○	○	○

Page 6: Final page

Thank you for your time completing this survey.

We are keen to hear as many opinions as possible across different UK units and therefore we would be grateful if you are the lead specialist nurse at your unit if you could forward this survey to your fellow nursing colleagues on your team.

Should you require any further information or have any questions please contact the project team via email
(Christopher.Wallace3@nhs.net or Greig.Taylor@newcastle.ac.uk)

Many thanks,

Project Team

Mr. Chris Wallace- StR in Paediatric Dentistry

Mr. Greig Taylor- NIHR Doctoral Research Fellow/StR in Paediatric Dentistry

Dr. Chris Vernazza- Senior Lecturer/Hon. Consultant in Paediatric Dentistry

Dr. Nidhi Singhal- StR in Paediatric Nephrology

Ms.Victoria Emmet- Specialist Nurse

Dr. Yincent Tse- Consultant in Paediatric Nephrology

Dr. Vijaya Sathyanarayana- Consultant in Paediatric Nephrology
